# Transcription Factors and Methods for the Pharmacological Correction of Their Activity

**DOI:** 10.3390/ijms26136394

**Published:** 2025-07-02

**Authors:** Svetlana V. Guryanova, Tatiana V. Maksimova, Madina M. Azova

**Affiliations:** 1 M.M. Shemyakin and Yu.A. Ovchinnikov Institute of Bioorganic Chemistry, Russian Academy of Sciences, 117997 Moscow, Russia; 2 Medical Institute, Peoples’ Friendship University of Russia, 117198 Moscow, Russia

**Keywords:** NF-κB, Nrf2, p53, STATs, HIF-1α, AP-1, PROTACs, molecular glues, epigenetic modulators, inhibition, tolerance

## Abstract

Transcription factors (TFs) are proteins that control gene expression by binding to specific DNA sequences and are essential for cell development, differentiation, and homeostasis. Dysregulation of TFs is implicated in numerous diseases, including cancer, autoimmune disorders, and neurodegeneration. While TFs were traditionally considered “undruggable” due to their lack of well-defined binding pockets, recent advances have made it possible to modulate their activity using diverse pharmacological strategies. Major TF families include NF-κB, p53, STATs, HIF-1α, AP-1, Nrf2, and nuclear hormone receptors, which take part in the regulation of inflammation, tumor suppression, cytokine signaling, hypoxia and stress response, oxidative stress, and hormonal response, respectively. TFs can perform multiple functions, participating in the regulation of opposing processes depending on the context. NF-κB, for instance, plays dual roles in immunity and cancer, and is targeted by proteasome and IKKβ inhibitors. p53, often mutated in cancer, is reactivated using MDM2 antagonist Nutlin-3, refunctionalizing compound APR-246, or stapled peptides. HIF-1α, which regulates hypoxic responses and angiogenesis, is inhibited by agents like acriflavine or stabilized in anemia therapies by HIF-PHD inhibitor roxadustat. STATs, especially STAT3 and STAT5, are oncogenic and targeted via JAK inhibitors or novel PROTAC degraders, for instance SD-36. AP-1, implicated in cancer and arthritis, can be inhibited by T-5224 or kinase inhibitors JNK and p38 MAPK. Nrf2, a key antioxidant regulator, can be activated by agents like DMF or inhibited in chemoresistant tumors. Pharmacological strategies include direct inhibitors, activators, PROTACs, molecular glues, and epigenetic modulators. Challenges remain, including the structural inaccessibility of TFs, functional redundancy, off-target effects, and delivery barriers. Despite these challenges, transcription factor modulation is emerging as a viable and promising therapeutic approach, with ongoing research focusing on specificity, safety, and efficient delivery methods to realize its full clinical potential.

## 1. Introduction

Transcription factors (TFs) are proteins that control gene expression by binding to specific DNA sequences and regulating transcription. Eukaryotic RNA polymerases are not able to bind to their respective promoters on their own. They rely on proteins called transcription factors, which regulate transcription but are not subunits of RNA polymerase [1]. TFs, by binding to specific DNA sequences, regulate cell specialization at all stages of development, starting with the embryonic stage, and subsequently control all homeostatic processes of the body [2,3,4]. It emerges that transcription factors specifically bind to many more sites than the number of genes that they dynamically regulate when measured; a transcription factor can have 10–100 times more binding sites throughout the genome than the number of genes that depend on this factor or respond to it [5,6]. Notably, site-specific TFs may be much more effective catalysts of change than gatekeepers of gene regulation [7].

Dysregulation of TF activity underlies many pathological processes, including malignancies, inflammatory diseases, and neurodegenerative disorders [8]. Normally, cells tightly control TF activity, but stress signals coming into the cell from its microenvironment, as well as internal signals such as genetic mutations, gene fusions, aberrant post-translational modification, etc., can lead to persistent activation or inactivation of these factors, contributing to the development of diseases. Historically, TFs have been considered “undruggable” drug targets due to their lack of convenient small molecule binding sites and largely disordered structure [9]. However, interest in therapeutic interventions for TFs has continued unabated. Recent years have seen the emergence of new approaches, including direct inhibitors, molecular glues, and protein targeting assays (PROTACs), which have demonstrated success in preclinical and clinical studies [9]. This review is devoted to pharmacological modulation of transcription factor activity. The review is broad in nature with an emphasis on the most studied TFs (NF-κB, A 20, p53, HIF-1α, STAT, etc.), their relationship with diseases (cancer, inflammation, neurodegeneration, etc.), current and promising approaches to regulating their activity, mechanisms of drug action, as well as the difficulties of developing highly specific and safe therapeutic agents.

## 2. Main Classes of Transcription Factors

TFs constitute a large and heterogeneous group of proteins classified by the structure of the DNA-binding domain and functional features.

### 2.1. Classification of Transcription Factors by Structural Complexity

Transcription factors control gene expression by binding specific DNA sequences and influencing transcription. Most TFs share a core architecture consisting of at least two components: a DNA-binding domain (DBD), which targets specific genomic sequences, and an effector domain, which activates or represses transcription. Beyond this basic plan, TFs vary widely in structural and regulatory complexity. TFs are classified into four broad classes based on domain architectures, increasing structural/functional complexity for each class.

#### 2.1.1. Class I Simple Transcription Factors (DBD + Effector Domain)

These are minimal TFs that typically consist of a single polypeptide requiring only a DNA-binding domain and an activation or repression effector domain. They often function as monomers or simple dimers and do not contain additional specialized regulatory domains. The effector (activation/repression) domain directly interacts with the transcriptional machinery or chromatin to modulate gene expression. Regulation of simple TFs is usually straightforward—for example, via small molecule binding or simple allosteric changes—without elaborate multi-step signaling cascades. Some TFs also have a relatively simple two-domain design. For example, the yeast GAL4 protein has a N-terminal Zn(II)_2Cys_6 binuclear cluster DNA-binding domain and a C-terminal acidic activation domain, with minimal other domains [10]. Similarly, the human Sp1 transcription factor contains a DNA-binding domain composed of zinc finger motifs and distinct glutamine-rich activation domains, but essentially relies on just these domains to function [11]. Homeodomain proteins (e.g., Hox factors) provide another example: they have a single helix-turn-helix DNA-binding domain (the homeodomain) and an intrinsically disordered activation domain [12]. These simple eukaryotic TFs often function by directly recruiting the basal transcriptional machinery or co-activators via their effector domains. Regulation can involve concentration, localization, or availability of a ligand, but generally Class I TFs do not require complex multicomponent signaling for activation. For instance, many are constitutively nuclear and active once expressed, or are modulated by a small ligand/hormone or a simple post-translational modification of the effector domain. Because Class I factors have only the core DBD and effector modules, their control is often at the level of DNA binding affinity or effector domain activity. They exemplify the fundamental paradigm that a TF’s DBD targets it to specific genes, and the effector domain then either recruits RNA polymerase (if an activator) or blocks it or recruits repressors (if a repressor).

#### 2.1.2. Class II: Intermediate/Modular Transcription Factors

Intermediate TFs possess more complex, modular architectures than the simple two-domain proteins. They often contain multiple distinct domains beyond the basic DBD and activation/repression domain—for example, dimerization interfaces, multiple DNA-binding modules, or domains for protein–protein interactions with co-regulators. They may function as multi-subunit complexes (obligate dimers or heterodimers), and their activity can be regulated by post-translational modifications or interactions that modulate these additional domains. In other words, Class II factors are modular: they assemble multiple functional pieces (within one protein or via subunit partnerships) to achieve regulation, but they generally do not require large signaling cascades or cytoplasmic sequestration for activation.

The AP-1 transcription factor (activator protein 1) exemplifies an intermediate TF. AP-1 is typically a heterodimer of Fos and Jun proteins, each of which has a basic leucine zipper (bZIP) domain for DNA binding and dimerization [13]. The bZIP domain (60–80 amino acids) combines a DNA-binding basic region with an adjoining leucine zipper α-helix that mediates Fos–Jun dimerization [14]. In addition, Jun and Fos have N-terminal transactivation domains that can be modulated by phosphorylation; for example, c-Jun’s N-terminal domain is phosphorylated by JNK kinases, enhancing its activity [15]. Thus, AP-1 requires both subunits to dimerize (via a protein–protein interaction domain) in order to bind DNA, and its activity is fine-tuned by kinase signaling that targets its activation domain, but it is not sequestered in an inactive complex in the cytoplasm. Another example is the tumor suppressor p53, a sequence-specific TF that is highly modular. p53 is a 393-aa protein with five domains: an N-terminal transactivation domain a proline-rich regulatory segment, a central DNA-binding core domain 3 that recognizes specific DNA motifs, a tetramerization domain required for forming the p53 homo-tetramer, and a C-terminal regulatory domain involved in down-regulating DNA binding [16]. p53 must form a tetramer via its oligomerization domain to bind DNA with full affinity, and its activity is controlled by various post-translational modifications (phosphorylation, acetylation) on its N- and C-termini, as well as binding of negative regulators like Mdm2 [17]. These features place p53 in the intermediate class—it has multiple domains and complex regulation. It is kept inactive by Mdm2-mediated degradation until stress signals modify p53, but it does not require a dedicated cytosolic inhibitor or multi-step cascade for nuclear entry; p53 is constitutively nuclear and DNA-bound, albeit kept in check by Mdm2.

Thus, in Class II TFs, regulation often involves protein–protein interactions or covalent modifications that modulate the TF’s multi-domain structure. Dimerization is a common requirement; for example, bZIP and basic helix-loop-helix (bHLH) factors must dimerize to bind DNA. The presence of dedicated dimerization domains, like leucine zippers or HLH motifs, means these TFs can integrate regulatory signals by controlling dimer assembly. Many intermediate TFs are also targets of signaling pathways via phosphorylation. For example, phosphorylation of c-Jun on Ser63/73 in its activation domain enhances AP-1 transcriptional activity, and phosphorylation of p53 on N-terminal sites disrupts Mdm2 binding and stabilizes p53 [18]. Unlike the “complex” class below, these modifications typically modulate the TF’s activity or stability within the nucleus or the ability to form the active DNA-binding complex, rather than gate the TF’s cellular localization. In summary, Class II TFs have modular domain architecture and are frequently regulated by assembly and PTMs, but they generally become active without needing an entirely separate inhibitor protein to be removed or a membrane-to-nucleus relay; they respond to signals more directly, often by a single-step modification or partner binding event.

#### 2.1.3. Class III: Complex Signal-Regulated TFs

This class encompasses transcription factors that are kept in an inactive state through association with inhibitory proteins or retention in the cytoplasm, and that require multi-step signaling cascades, often involving kinase activation and protein degradation or cleavage for their activation. These TFs usually have multiple subunits or domains, and their activation is tightly controlled by cellular signaling pathways, such as those triggered by cytokines, growth factors, or stress signals. Complex TFs often serve as endpoints of signaling cascades, integrating extracellular stimuli into nuclear transcriptional responses. A hallmark of this class is regulated nuclear entry; the TF is usually sequestered outside the nucleus until the appropriate signal is received.

The NF-κB family of transcription factors illustrates this complexity of activation. In resting cells, NF-κB dimers, typically a p65/RelA–p50 heterodimer, are held inactive in the cytoplasm by inhibitory IκB proteins, which mask the NF-κB nuclear localization signals. Thus, NF-κB exists as a latent cytosolic complex under basal conditions [19]. Upon cell stimulation, for example, by inflammatory cytokines like TNFα or by pathogen-associated signals), a kinase cascade is triggered. The IκB kinase (IKK) complex becomes activated and phosphorylates the IκB inhibitor. Phosphorylated IκB is then ubiquitinated and targeted for proteasomal degradation. This degradation frees the NF-κB dimer, exposing its nuclear localization sequence (NLS). The NF-κB dimer subsequently translocates into the nucleus, where it binds κB enhancer sequences and induces target gene expression. NF-κB activation requires a signaling cascade that connects an upstream sensor, for example, a TNF receptor to IKK activation, IκB destruction, and finally NF-κB nuclear import. In this way, NF-κB activation is a much more elaborate control than seen in Classes I or II. NF-κB’s structure reflects this regulation. NF-κB subunits (Rel family proteins) each have an N-terminal Rel Homology Domain (RHD) responsible for dimerization, DNA-binding, and IκB binding, and some (like p65) carry C-terminal transactivation domains. Two NF-κB subunits form a dimer that is inhibited by one IκB, which contains ankyrin repeat domains binding the RHD. Only after IκB is removed by the signaling pathway does the dimer act as a transcription factor [20].

Several other TFs fit the paradigm of latent cytosolic factors activated by signaling. STAT proteins (Signal Transducers and Activators of Transcription) are latent monomers in the cytoplasm that become activated through tyrosine phosphorylation by receptor-associated Janus kinases (JAKs). Upon cytokine stimulation, a STAT is phosphorylated, dimerizes via its SH2 domain, and then translocates to the nucleus to regulate gene expression; thus STATs, like NF-κB, lie dormant in the cytoplasm until a signaling event occurs [21]. Another example are the SMAD proteins in TGF-β signaling. Receptor kinases phosphorylate R-Smad proteins, which then form complexes with Co-Smad (Smad4) and move into the nucleus as active TF complexes [22]. NFAT (Nuclear Factor of Activated T-cells) is yet another example, remaining phosphorylated and cytosolic until calcium/calcineurin signaling prompts its dephosphorylation and nuclear import. All of these TFs share a reliance on post-translational modification and controlled localization; they often contain regulatory domains for phosphorylation sites or interaction motifs for inhibitors that keep them inactive until the right signal. In terms of domain architecture, such factors may not be drastically larger than intermediate TFs, but their regulatory mechanisms are more complex, involving dedicated inhibitor proteins (IκB for NF-κB) or cytosolic tethering that is relieved only by specific signaling events [23].

Class III TFs act as convergence points of signaling pathways. They typically require phosphorylation by upstream kinases and sometimes proteolysis of inhibitors for activation. This adds layers of regulation: signal specificity, amplification, and opportunities for feedback control. For example, NF-κB activation is transient because newly made IκBα in the nucleus can shut off NF-κB and export it—an added level of feedback control [20]. Compared to Class II, these TFs are not constitutively able to bind DNA even if their DBD is intact; their cellular localization and activation state are tightly controlled by other factors. This complexity allows the cell to keep these potent TFs off until they are truly needed, preventing inadvertent gene activation. Overall, Class III factors underscore how cells employ intricate signaling circuits to regulate transcription factor activity in time and space.

#### 2.1.4. Class IV: Nuclear Receptors

Nuclear receptors (NRs) are a specialized family of eukaryotic transcription factors defined by a ligand-dependent activation mechanism and a conserved multi-domain structure. They typically function as hormone or metabolite sensors that directly bind lipophilic small-molecule ligands, such as steroids, thyroid hormone, retinoids, and, upon ligand binding, regulate gene expression [24]. Structurally, nuclear receptors have a characteristic domain architecture comprising an N-terminal variable region, often containing an activation function AF-1 domain, a highly conserved DNA-binding domain (C domain) with two zinc-finger motifs, a hinge region (D domain) that provides flexibility, and a large C-terminal ligand-binding domain (LBD, E domain), which often contains a ligand-dependent activation function (AF-2). Some NRs also have a short C-terminal extension (F domain) that is variable in presence and function. Nuclear receptors often form homo- or heterodimers and bind DNA at specific response elements, typically as dimers [25]. The steroid hormone receptors—the glucocorticoid receptor (GR), estrogen receptor (ER), androgen receptor (AR), and progesterone receptor—exemplify this class. GR, in the absence of hormone cortisol, is sequestered in the cytoplasm in an inactive complex with chaperone proteins, notably Hsp90 [26,27].

GR’s LBD binds the chaperone, and its DBD is not engaged in DNA. When hormone enters the cell and binds the GR LBD, the receptor undergoes a conformational change that causes it to dissociate from Hsp90 and expose its NLS. The ligand-bound GR dimer translocates into the nucleus, where its zinc-finger DBD binds to glucocorticoid response elements (GREs) in target gene promoters, and its activation domains recruit coactivator complexes to stimulate transcription. Similarly, the estrogen receptor α (ERα) resides largely in the nucleus, even without ligand, but is kept in an inactive state bound to corepressor proteins [28]. Binding of estrogen to the ER LBD induces a structural reconfiguration that releases corepressors and promotes the binding of coactivators, enabling transcription of estrogen-responsive genes. Other nuclear receptors, such as the thyroid hormone receptor (TR), form heterodimers with RXR and bind DNA constitutively, but switch from repressing to activating target genes upon ligand binding. Thyroid hormone causes dismissal of corepressors and recruitment of coactivators [29].

Despite some differences, all NRs share the principle of ligand-regulated transcriptional activation. The presence or absence of a small molecule ligand in the LBD acts as a molecular switch. In addition, many NRs can be modulated by phosphorylation and other PTMs that fine-tune their activity or alter their subcellular localization, but the ligand is the primary control [30].

The four classes above illustrate an increasing order of regulatory complexity, from simple one-component TFs to those integrated into elaborate signaling pathways. Class I and II factors provide direct, often immediate control of transcription, suitable for housekeeping and straightforward gene regulation, whereas Class III and IV factors allow multi-layered control, signal-dependent activation, and tight spatiotemporal regulation, suitable for responsive genes in signaling networks.

### 2.2. Classification of Transcription Factors by Functional Activity

Examples of major families include NF-κB (Rel family), tumor suppressor p53 (p53 family), STAT factors (STAT1-STAT6 family), HIF (HIF-1α/ARNT heterodimer, bHLH-PAS family), AP-1 (c-Fos/c-Jun dimer, bZIP family), nuclear hormone receptors (ligand-bound receptors for steroid hormones, retinoids, etc., containing a zinc-finger DNA domain), and Nrf2 (CNC-bZIP family). These factors control key cellular processes: NF-κB is responsible for the immune response and inflammation; p53 is responsible for DNA repair, cell cycle arrest, and apoptosis (often inactivated in cancer); STATs transmit cytokine signals to the nucleus, regulating immune and oncological processes; HIF-1α controls adaptation to hypoxia and is a critical transcription factor for B cells producing IL-10 in autoimmune diseases; AP-1 is involved in the regulation of cell proliferation and stress response; Nrf2 controls antioxidant defense, autophagy, and mitochondrial biogenesis; nuclear receptors mediate the action of hormones [1,31,32,33,34]. Disruptions in the functioning of these factors are associated with many diseases; for example, hyperactivation of NF-κB is observed in autoimmune diseases and cancer, mutations in the TP53 gene occur in more than half of cancer cases, excessive HIF-1α activity promotes tumor growth under hypoxic conditions, and STAT3 imbalance is detected in malignant neoplasms and chronic inflammation [8,9]. Table 1 summarizes some of the best-studied TF classes, their functions, and associated pathological conditions.

## 3. NF-κB

NF-κB is a master regulator of innate and adaptive immunity and inflammatory responses, first discovered in B lymphocytes, and binding to the enhancer of the immunoglobulin light chain encoding encode [35,36]. Currently, the involvement of NF-κB in inflammation and immune responses is considered to be the most important role that NF-κB signaling plays in biology [19]. Initially discovered in humans, NF-κB has been identified in other mammals as well as insects [37]. NF-κB homologs and many upstream signaling components have also been characterized in basal phyla, including *Cnidaria* (sea anemones, corals, hydras, and jellyfish), *Porifera* (sponges), and protozoa [38,39,40,41]. NF-κB polymorphisms underlie many diseases, including autoimmune, neurodegenerative, and oncological diseases [42]. NF-κB transcription factors consist of hetero- or homodimers of proteins—NF-κB1 (or p50), NF-κB2 (or p52), RelA (or p65), RelB, and c-Rel—and can be activated in both classical and non-canonical ways.

The classical activation pathway depends on IKKβ and IκB degradation and is activated by various cytokines, ROS, TLR, and NLR ligands [43,44,45,46,47]. NF-κB integrates external signals and regulates many vital processes; knockout of NF-κB causes death at the embryonic stage of development [48,49]. The classical active NF-κB complex is a p65/p50 heterodimer retained in the cytoplasm by the IκB inhibitor. Under the influence of stimuli (e.g., cytokines, microbial products, or stress), IκB is phosphorylated by the IKK kinase complex, and undergoes ubiquitination and proteasomal degradation, releasing NF-κB for translocation into the nucleus and activating cytokine genes, survival molecules, etc. [50]. Normally, canonical NF-κB activation is a host defense response to eliminate the pathogen [8,51]. On the other hand, canonical NF-κB activation promotes proliferation, survival, angiogenesis, and invasion of tumor cells, contributing to tumor promotion and progression [50,52].

Canonical NF-κB is activated following activation of pattern recognition receptors (PRRs), including toll-like receptors (TLRs), RIG-I-like receptors, NOD-like receptors (NLRs), C-type lectin-like receptors, and cytosolic DNA sensors [53,54,55]. Moreover, the main classes of PRRs that are predominantly involved in the recognition of molecular structures unique to microbial pathogens are considered to be Toll-like receptors (TLRs) and nucleotide oligomerization domain-like receptors (NODs) [55,56,57]. TLRs recognize lipopolysaccharides, which can have a wide variety of structures, and NODs recognize muramyl peptides [56,57,58,59,60,61,62]. Canonical NF-κB is also activated by DNA damage, which causes activation of ataxia telangiectasia mutated and a series of sequential post-translational modifications of NEMO in the nucleus, including SUMOylation, phosphorylation, and ubiquitination [63,64]. Pathologically increased NF-κB activity contributes to chronic inflammation, rheumatoid arthritis, inflammatory bowel disease, and tumor cell resistance to apoptosis, which is observed in many types of cancer. The role of canonical NF-κB in cancer is complex, with both positive and negative roles in inflammation, cancer initiation, and progression [43,65,66,67,68,69,70,71].

Interestingly, the dynamic change in NF-κB depends on the nature of the stimulus, its duration, and the combination of different stimuli. Long-term exposure to stimuli may induce cross-tolerance between multiple stimuli to prevent immune-mediated damage, or may enhance subsequent responses to facilitate pathogen clearance through priming or immune training [72,73]. Dysregulation of NF-κB may promote cancer progression by controlling epithelial-mesenchymal transition and metastasis. NF-κB is often associated with upregulation of matrix metalloproteinases (MMPs), which act on the extracellular matrix to promote tumor cell evasion. Additionally, NF-κB may promote tumor progression by upregulating vascular endothelial growth factor (VEGF) and its receptors [74].

The noncanonical (alternative) NF-κB pathway is mediated by RelB/p52 heterodimers and is regulated by the NIK-IKKα axis [75,76,77]. p100 is a precursor of p52 that functions to preferentially block RelB translocation to the nucleus. Partial proteolysis of p100 produces p52 and releases RelB, forming a RelB/p52 dimer that translocates to the nucleus. Thus, targeting p100 is a central event in the non-canonical NF-κB signaling pathway. Constitutively, p100 is only slightly converted to p52. Processing of p100 is inhibited by its C-terminal processing inhibitory domain (PID) and ankyrin repeat domain (ARD). PID has a death domain (DD), and mutation in DD abolishes the inhibitory function of PID [76,78]. The alternative NF-κB pathway is activated by limited stimuli and is critical for stromal cell function and maturation, B cell activation and survival, and lymphoid organ development and adaptive immunity [79,80,81,82,83,84,85,86]. On the other hand, hyperactivity of non-canonical NF-κB is associated with malignancies such as multiple myeloma and lymphoma [87,88,89,90]. One reason may be that mutation of the upstream signaling molecules TRAF3 or cIAP leads to abnormal accumulation of NIK and activation of non-canonical NFκB, leading to multiple myeloma [79]. On the other hand, deletion of NIK suppresses tumor formation in a mouse model [87]. In addition, rearrangements or mutations of NF-κB2 leading to sequential non-canonical activation of NF-κB have also been found in various human malignancies, including multiple myeloma, and T-cell and B-cell lymphoma [91,92].

NF-κB has been shown to play a role in neurodegenerative diseases, for example, in the development of Alzheimer’s disease (AD), Parkinson’s disease (PD), amyotrophic lateral sclerosis (ALS), and multiple sclerosis (MS). Its activation in neurons and glial cells can have both neuroprotective and neurotoxic effects, depending on the context, cell type, and binding partner in the heterodimer translocating to the nucleus [93,94,95,96]. When studying the effect of two opposing modulators of cell viability, IL-1beta and glutamate, it was found that IL-1beta activated the p50, p65, and c-Rel subunits of NF-κB, whereas glutamate activated only the p50 and p65 proteins [97]. Stimulation of cells with glutamate correlated with the expression of the proapoptotic genes Caspase-3, Caspase-2L, and Bax. In contrast, IL-1beta induced the expression of the short antiapoptotic isoform of Caspase-2 [97]. Thus, the RelA subunit of the activated p50/RelA dimer plays a key role in the onset of neurodegenerative processes induced by ischemic strokes, as well as glutamate or beta-amyloid toxicity, while another c-Rel subunit in activated NF-κB dimers determines the resistance of neurons to brain strokes [83,97,98,99,100]. Constitutive knockout of c-Rel disrupts the resistance of dopaminergic (DA) neurons of the substantia nigra (SN) to aging and causes parkinsonism-like pathology in mice [100]. NF-κB can be activated in microglia by α-synuclein, which leads to chronic inflammation and death of dopaminergic neurons in the substantia nigra [101]. In patients with PD, the proportion of dopaminergic neurons with immunoreactive NF-κB in their nuclei was more than 70-fold higher than in control subjects [102].

Thus, NF-κB activation in microglia and astrocytes leads to the production of proinflammatory cytokines (TNF-α, IL-1β, and IL-6), which enhances neuroinflammation and promotes the accumulation of β-amyloid (Aβ) and tau protein, while in neurons, NF-κB can have a protective effect by promoting cell survival through the activation of anti-apoptotic genes (Bcl-2, Bcl-xL) [94,100,101,102,103].

### Pharmacological Modulation

Both activation and inhibition of NF-κB are of pharmacological importance. Activation of NF-κB is relevant in vaccination, in case of insufficient immune response in chronic infectious diseases, and in the treatment of some allergic diseases with insufficient activity of innate immunity [104,105,106,107]. Others promising treatments include selective inhibition of NF-κB [108]. There are various approaches to inhibiting the NF-κB pathway. An indirect but clinically successful method is proteasome blockade; the drug bortezomib, which reversibly inhibits the 26S proteasome, prevents the degradation of IκBα and thereby suppresses NF-κB activation [109,110,111]. Bortezomib is approved for the treatment of multiple myeloma, where NF-κB promotes the survival of tumor plasma cells. However, NF-κB suppression is associated with immunosuppression; for example, long-term bortezomib therapy increases the risk of infections due to the inhibition of both the canonical and alternative NF-κB pathways [112,113,114].

Another approach is direct inhibition of the IKKβ kinase, which is responsible for the phosphorylation of IκB; Small molecules, such as SDX-308, MLN120B, and BMS-345541, that selectively inhibit IKKβ and thereby block NF-κB activation have been developed [115,116,117,118,119]. Broad-spectrum anti-inflammatory drugs also act in part through inhibition of NF-κB. Corticosteroids (for example, dexamethasone) induce IκBα synthesis and salicylates (aspirin) directly inhibit IKKβ activity [120,121,122]. Aspirin, salicylic acid, and glucocorticoids have been shown to inhibit NF-κB activation, although this may cause immunosuppression with long-term use [72]. Experimental strategies also include decoy oligonucleotides—short DNAs that distract NF-κB from binding to target genes—as well as peptides that mimic regions of IκB (to retain NF-κB in the cytoplasm) [123]. Despite the many options for inhibiting NF-κB, the therapeutic benefit must outweigh the risks. Probably the most promising option is short-term suppression of NF-κB or a combination of its inhibitors with other methods of treating tumors, since the blockade of NF-κB alone may not be sufficient for complete tumor regression.

When pharmacologically targeting the NF-κB signaling pathway, it is also important to consider that NF-κB plays an important role in the immune response, and long-term use of NF-κB inhibitors can lead to immunodeficiency [72,124]. In addition, a combination of NF-κB inhibitors and traditional treatments is necessary, since blocking NF-κB alone may not be sufficient for effective therapy [72]. NF-κB modulation is considered a promising therapeutic strategy; however, due to the broad involvement of NF-κB in physiological immune processes, its use is associated with risks.

## 4. p53

p53 is a “guardian of the genome”, a classic tumor suppressor transcription factor. Normally, p53 remains at a low level due to constant degradation; E3-ubiquitin ligase MDM2 binds to p53 and marks it for destruction [125,126]. Under the influence of stress, DNA damage, or oncogenic signals, p53 is stabilized and activates the transcription of genes that arrest the cell cycle (p21) or trigger apoptosis (PUMA, NOXA). TP53 mutations are the most common genetic defect in cancer, leading to the loss of p53 function or the acquisition of oncogenic properties [127]. In cells with wild-type p53, its function is often suppressed by MDM2/MDMX hyperactivity or viral oncoproteins [128]. Therefore, restoring p53 function is an attractive target for cancer therapy [127,129,130].

### Pharmacological Modulation

The main strategy is to activate p53 in tumor cells. This is achieved by disrupting the interaction of p53 with MDM2. Small molecules of the Nutlin class, such as Nutlin-3, were the first selective inhibitors of the p53–MDM2 linkage [131]. Nutlin-3 and its improved derivatives (e.g., RG7112, DIMP53-1, etc.) occupy the hydrophobic groove on MDM2, mimicking the key amino acids of p53, and prevent p53 ubiquitination [130,131,132,133]. This leads to the stabilization of p53, the accumulation of active p53 in the nucleus, and the initiation of target gene transcription, cell cycle arrest, and apoptosis in cells with intact TP53 [129]. This approach has proven its feasibility in principle. Small Nutlin molecules became the first evidence that disruption of the p53–MDM2 interaction can have an antitumor effect [129,134]. Some MDM2 inhibitors have reached clinical trials; for example, idasanutlin (RG7388) has been studied in leukemia and solid tumors [135,136,137,138].

Another approach is the reactivation of mutant p53. The compound APR-246 (PRIMA-1^Met) is able to covalently bind to mutant p53 via cysteine residues, partially restoring its native conformation and the function of the transcription factor [139]. APR-246 has passed phase I/IIa clinical trials, demonstrating its ability to restore tumor suppression in some mutant p53 variants [139,140]. Another approach is peptide mimetics; for example, “stapled” α-helical peptides have been developed that mimic the region of p53 bound by MDM2/MDMX, which allows the blocking of these p53 inhibitors. An example is the drug ALRN-6924 (Stapled Peptide), currently in clinical trials, which binds to MDM2/MDMX and prevents their interaction with p53, thereby activating the p53 pathway in cancer cells [141,142,143].

In special cases, the opposite is possible. For the inhibition of p53, for example, the compound pifithrin-α was studied for its role in protecting normal cells from radiation by temporarily suppressing p53-dependent apoptosis. However, the main focus is on p53 activation in cancer therapy. It should be noted that, due to the functions of p53 in normal cells, over activation of this factor is fraught with toxicity, for example, bone marrow damage, so MDM2 inhibitors often cause hematological side effects. In addition, ALRN-6924 showed promise in the prevention of myelotoxicity in patients whose cancer has deleted or mutated TP53, but did not demonstrate any significant activity in a randomized controlled trial [144]. However, combination therapy (p53 activation plus inhibition of tumor survival pathways) is considered a promising direction in oncology [142,143,144]. Despite numerous studies of MDM2–p53 inhibitors, not a single drug has entered the market [145]. However, currently more than 10 compounds are being evaluated in the clinic, both as single agents and in combination with other targeted therapies, including two inhibitors in phase 3 studies and two compounds that have received orphan drug/fast track designation from the FDA [145]. Moreover, the p53 family of TFs and its regulators offer endless possibilities for cancer therapy, and a systems approach, using modern systems biology and bioinformatics methods, improves the implementation of precision medicine [146,147,148,149].

## 5. HIF-1α

HIF-1 (hypoxia-inducible factor 1) is the main transcriptional regulator of the hypoxia response. It is a heterodimer of the oxygen-sensitive subunit HIF-1α and the constitutive HIF-1β (ARNT) [129]. In normoxia, HIF-1α is rapidly destroyed. Proxyl hydroxylase enzymes (PHD1-3) hydroxylate HIF-1α, which leads to its interaction with von Hippel–Lindau ubiquitin ligase (pVHL) and proteasomal degradation. In hypoxia, hydroxylation ceases, and HIF-1α accumulates and migrates to the nucleus, where it dimerizes with HIF-1β, activating genes that provide adaptation to low O_2_, angiogenesis—VEGF, erythropoiesis—EPO, glycolysis, etc. [150]. In solid tumors, rapid proliferation creates a microenvironment with a lack of oxygen, which forces tumor cells to restructure their metabolism and genetic instability, and resistance to chemotherapy appears [151]. Tumors adapt to hypoxia, metabolic reprogramming and stimulation of neovascularization occur [151,152,153]. In pathological conditions, excessive HIF-1α activity promotes cancer progression and fibrosis [152,153].

In contrast, HIF enhancement may be useful in ischemic diseases and anemia for tissue protection. In particular, in neurodegenerative diseases, a decrease in HIF-1a levels is observed, which is associated with the increased phosphorylation of tau protein and the formation of neurofilaments in Alzheimer’s disease, and the introduction of HIF-1 has a protective effect [154]. Enhancement of HIF-1α activity is considered a central strategy for the treatment of cerebral ischemia [155].

### Pharmacological Modulation

Depending on the context, the goal may be either inhibition or activation of HIF. HIF-1α inhibitors are being studied as potential anticancer agents. One direct approach is to disrupt the dimerization of HIF-1α with HIF-1β. The small molecule acriflavine, previously used as an antiseptic, has been identified as a potent inhibitor of HIF-1 dimerization. It binds to the PAS-B domain of HIF-1α/2α and prevents their interaction with HIF-1β, thereby reducing the transcription of hypoxic response genes and inhibiting tumor growth and vascularization [156,157]. Acriflavine has shown efficacy in preclinical models of melanoma and other cancers, but its clinical use is limited by toxicity; nanocapsules for targeted delivery of acriflavine are being developed to reduce side effects [156,158]. Another approach is the inhibition of HIF binding to DNA; anthracycline antibiotics doxorubicin and eunomycin intercalate into DNA and prevent HIF-1 activity from attaching to sites in promoters, suppressing the expression of VEGF genes and other HIF targets [159,160,161]. In addition, the CBP/p300 (HIF coactivator) inhibitor, the compound chetomin, interferes with the interaction of HIF with the transcriptional apparatus [162]. On the other hand, HIF activators have found applications in the treatment of anemia in chronic disease. A group of oral drugs, HIF prolyl hydroxylase inhibitors (PHD), imitate the state of hypoxia, stabilizing HIF-α and enhancing endogenous erythropoietin production in the kidneys. For example, roxadustat, daprodustat, and a number of analogues are approved for the treatment of chronic renal anemia; they block PHD enzymes and prevent degradation of HIF-1α/2α, which leads to an increase in hemoglobin levels [163,164]. Such treatment allows the use of recombinant erythropoietin to be avoided. At the same time, there are concerns that long-term activation of HIF-PHD inhibitors can affect oncogenesis or cause cardiovascular side effects and thrombotic complications, so their safety is carefully studied [163,164,165].

Thus, HIF-1α is of dual interest as a target for inhibition in oncology and as a target for activation in ischemic conditions. Selective HIF-2α inhibitors, which are important in renal cell carcinoma, are in development, as are combination approaches, for example, combining HIF inhibitors with antiangiogenic drugs.

## 6. STAT (STAT1/3/5)

The Signal Transducers and Activators of Transcription (STAT) family includes seven factors which transmit the signal from cytokine receptor activation to the nucleus. Upon the binding of a cytokine (interleukins, interferons, or growth factors), the receptor activates the associated tyrosine kinase of the JAK family, which phosphorylates cytoplasmic STATs. Phosphorylated STATs form dimers, translocate to the nucleus, and induce the transcription of immune response genes, proliferation, and differentiation [166,167,168].

STAT3 is the most well-known oncogenic member of the family, constitutively active in many solid tumors and lymphomas, promoting the expression of survival genes and inflammatory mediators [169,170,171].

STAT5 is involved in the pathogenesis of leukemia upon activation of tyrosine kinase oncogenes and is considered a key protein in erythropoietin signaling [172,173,174]. STAT1 and STAT2 are critical for antiviral immunity via IFN signaling [175,176]. Over activation of the JAK/STAT pathway contributes to autoimmune diseases, such as increased IL-6/STAT3 signaling in rheumatoid arthritis [169]. Thus, STAT regulation is relevant in both oncology and inflammatory pathologies.

### Pharmacological Modulation

Direct inhibition of STATs has long been a challenge, but interesting approaches have been developed. In small molecule STAT3 inhibitors, the first generation—compounds such as Stattic, STA-21, and S3I-201—block the SH2 domain of STAT3, preventing dimerization and phosphorylation [177,178,179]. They are used as in vitro toolkits, but have limited specificity and pharmacokinetics. A dihydroimidazolone derivative, napabukasin (BBI-608), has been tested in clinical trials to reduce the activity of STAT3-dependent genes in cancer cells; however, its mechanism is not entirely clear, and it is believed to act as a proxy killer of STAT3-activated cells [180,181,182].

JAK kinase inhibitors, which indirectly inhibit the entire JAK/STAT pathway, have achieved significant success. For example, tofacitinib, baricitinib, and upadacitinib, selective oral inhibitors of JAK1/3 or JAK1/2, are approved for the treatment of rheumatoid arthritis and other inflammatory diseases [158,183,184,185,186,187].

These drugs occupy the ATP-binding site of JAK kinases, preventing STAT phosphorylation, and have shown high efficacy in suppressing immunopathology. However, JAK inhibitors carry a risk of side effects (infections, thrombosis) due to the breadth of affected cytokine pathways [158,186,187,188,189,190,191]. Recently, fundamentally new methods have emerged, including degradation of STAT proteins using PROTACs. PROTACs—Proteolysis Targeting Chimeras—are hybrid molecules, one part of which bind to the target protein, for example, STAT3, and the other to the E3 ligase, for example, CRBN, which leads to ubiquitination and destruction of the target protein by the proteasome [192]. In 2019, the creation of PROTAC SD-36, which causes rapid and selective degradation of STAT3, was reported [191]. SD-36 consists of a ligand to the SH2 domain of STAT3, linked by a linker to the ligand Cereblon (CRBN). In cell models of leukemia, SD-36 completely abolishes STAT3, suppressing the expression of its targets and arresting tumor cell growth [189]. Importantly, SD-36 showed high selectivity for STAT3 over other family members, despite the homologous SH2 domain, and also caused tumor regression in mouse models with minimal toxicity [190,191].

This result demonstrates the advantage of protein degradation over simple inhibition of function; by completely abolishing STAT3, PROTACs also disable the non-transcriptional functions of STAT3 (directly in mitochondria, etc.), which could be preserved using inhibitors alone [190,191,192]. Based on these data, the PROTAC strategy is considered promising for “inaccessible” factors such as STAT3 [192].

## 7. AP-1

AP-1 (activator protein-1) is a general name for a family of dimeric factors consisting of the Jun (c-Jun, JunB, and JunD) and Fos (c-Fos, FosB, and Fra-1/2) proteins. AP-1 is activated in response to multiple signals (mitogens, stress, and cytokines) and regulates genes associated with proliferation, differentiation, and inflammation [193]. AP-1 binds to distal enhancers and remodels chromatin to maintain chromatin accessibility [194]. Increased AP-1 activity is observed during tumor growth; for example, overexpression of c-Fos and c-Jun in tumors is associated with cancer aggressiveness, and in chronic inflammation AP-1 stimulates cytokines and metalloproteinases [195,196]. In rheumatoid arthritis, a correlation between AP-1 levels in the synovium and disease severity has been found [169]. Thus, AP-1 inhibition is of interest as an antitumor and anti-inflammatory approach.

### Pharmacological Modulation

Direct targeting of AP-1 has been made possible by the development of a small molecule inhibitor, T-5224, which selectively binds to the c-Fos/c-Jun complex and prevents its interaction with DNA without affecting other factors [197].

T-5224 suppresses the expression of multiple proinflammatory genes (IL-1β, IL-6, TNF, and MMP) in arthritis models [169]. In animal studies, it improved the course of arthritis, and in phase II clinical trials, it showed safety in rheumatoid arthritis [198]. Although there are no approved drugs that directly target AP-1, as of 2025, T-5224 and other experimental inhibitors such as SR11302, which reduces AP-1 activity via the APP pathway, demonstrate the principle feasibility of such therapy. Indirectly, AP-1 activity can be reduced by blocking JNK or p38 MAPK kinases, which are required to phosphorylate AP-1 components. For example, a JNK inhibitor, SP600125, and p38 inhibitors reduce the level of active AP-1 and attenuate the production of inflammatory factors [169]. In addition, some anti-inflammatory agents, such as the phosphodiesterase 4 (PDE-4) inhibitor roflumilast, reduce AP-1 activity indirectly through increased cAMP and prevent the secretion of proinflammatory cytokines such as IL-6, IL-8, and TNF-α [199].

Prospects for targeting AP-1 are associated with combination therapy, for example, the simultaneous suppression of NF-κB and AP-1 for more complete blocking of inflammation. Another approach is the search for more potent and stable inhibitors of Fos/Jun protein–protein interactions.

## 8. Nrf2

Nrf2 (nuclear factor erythroid 2-like 2) is the main regulator of antioxidant protection and detoxification. In the absence of stress, Nrf2 associated with the cytosolic protein Keap1 is constantly ubiquitinated and destroyed. In the presence of oxidative or electrophilic stress, key cysteines of Keap1 are modified, which prevents Nrf2 degradation. Free Nrf2 accumulates and translocates to the nucleus, where it forms a heterodimer with Maf proteins and initiates the transcription of genes encoding antioxidant enzymes HO-1, NQO1, GST, proteasomal proteins, and xenobiotic efflux systems. Thus, Nrf2 protects cells from damage by reactive oxygen species and toxins [200,201]. In the clinic, activation of the Nrf2 pathway is beneficial in inflammatory and neurodegenerative diseases to reduce oxidative stress, such as multiple sclerosis and Parkinson’s disease [202,203,204]. However, in oncology, chronic hyperactivation of Nrf2 in tumor cells can lead to chemoresistance and tumor growth [205]. For example, biallelic inactivation of KEAP1 is a common genetic alteration in non-small cell lung cancer [206]. Nrf2 has been shown to have a dual role in carcinogenesis: protection at early stages, but tumor support at later stages [207].

### Pharmacological Modulation

The strategy of Nrf2 activation is reflected in the drugs already in use. A striking example is dimethyl fumarate (DMF), approved for the treatment of relapsing multiple sclerosis and psoriasis [208,209]. DMF is an electrophile and covalently modifies the thiol groups of Keap1, which leads to the dissociation of the Keap1–Nrf2 complex and translocation of Nrf2 into the nucleus [187,188,189,190]. As a result, DMF enhances the transcription of antioxidant genes and exhibits neuroprotective and anti-inflammatory effects [210,211]. Since DMF does not act directly on Nrf2, but on its inhibitor Keap1, it can be classified as an epigenetic/post-translational modulator of the pathway. Other small molecules with a similar mechanism, such as sulforaphane from cruciferous vegetables, omeprazole sulfide, and carbazoles, also modify Keap1 and activate Nrf2, and are being studied as potential cytoprotectors [210]. On the other hand, inhibition of Nrf2 may sensitize cancer cells to therapy if their Nrf2 pathway is overactive. Direct inhibitors of Nrf2 are few; approaches based on enhancing the interaction of Nrf2 with Keap1 and stabilizing the complex, as well as the suppression of Nrf2 synthesis, such as with antisense oligonucleotides or small molecules that reduce NFE2L2 transcription, are being explored [211,212,213,214]. One such approach is considered to be the inhibition of the Nrf2 clamp on ARE sequences by the factor MafK; however, there are no practically suitable compounds yet [215,216]. It should be emphasized that the activation of Nrf2 has already become a successful pharmacological strategy using DMF as an example, while the reverse modulation—inhibition—requires further research. Potentially, selective suppression of Nrf2 in tumors, for example, using gene therapy or nanodelivery of inhibitors, could enhance the effect of chemotherapy.

## 9. Nuclear Receptor Transcription Factors

Nuclear receptors (NRs) are a family of ligand-regulated transcription factors that control gene expression in key biological processes such as reproduction, development, and metabolism [217,218]. The family includes 48 receptors for steroids, thyroid hormones, and vitamins [219]. These receptors are evolutionarily conserved across metazoans; for example, the insect ecdysone receptor orchestrates metamorphosis, while vertebrate steroid (e.g., estrogen and androgen) and thyroid receptors govern sexual maturation and developmental transitions [220]. NRs typically possess a DNA-binding domain and a ligand-binding domain. Small hydrophobic molecules—including steroid and thyroid hormones, fat-soluble vitamins, fatty acids, and other lipids—serve as NR ligands, diffusing into cells to bind their cognate receptors. Ligand binding induces a conformational change in the NR that allows it to recognize specific DNA sequences and recruit transcriptional coregulators [221,222]. Through this ligand-dependent DNA binding and cofactor recruitment “switch”, NRs modulate downstream target genes that drive developmental programs and maintain metabolic homeostasis [223]. NR signaling is pivotal in disease. Dysregulation of nuclear receptors is implicated in hormone-dependent cancers and metabolic disorders [219,224,225]. Estrogen receptors (ER) and androgen receptors (AR) function as key drivers in many breast and prostate cancers, respectively, and their ligand-dependent activity underlies the success of endocrine therapies (e.g., anti-estrogens in ER^+^ breast cancer and anti-androgens in prostate cancer). Metabolic diseases such as type 2 diabetes and dyslipidemia are also linked to NR pathways [226]. For instance, peroxisome proliferator-activated receptor γ (PPARγ) governs adipogenesis and insulin sensitivity—the target of thiazolidinedione anti-diabetic drugs—whereas PPARα regulates fatty acid oxidation and serum lipid levels (targeted by fibrate drugs for hyperlipidemia) [224,227]. Given their ligand responsiveness, NRs are highly “druggable” nodes of signaling; indeed, an estimated 10–15% of FDA-approved drugs act on nuclear receptors [225].

### Pharmacological Modulation

Pharmacological ligands modulate NRs via agonism, antagonism, or selective modulation. Agonists stabilize an active conformation that recruits coactivators and activates transcription, whereas antagonists prevent coactivator recruitment to repress gene expression. Selective modulators induce intermediate receptor conformations, yielding tissue-specific transcriptional outcomes [225]. For example, tamoxifen, a selective ER modulator, antagonizes ER-driven transcription in breast cancer but exerts agonistic effects in uterine tissue [228]. Enzalutamide is an AR antagonist that blocks androgen receptor signaling in prostate cancer [229]. In metabolic disease, the PPARγ agonists thiazolidinediones activate adipocyte genes to improve insulin sensitivity [230,231]. LXR and FXR agonists, which regulate cholesterol and bile acid metabolism, are under investigation for atherosclerosis and non-alcoholic fatty liver disease [232]. Glucocorticoid agonists, for example, dexamethasone, are potent anti-inflammatory agents, and novel selective GR modulators are being developed to retain anti-inflammatory efficacy with reduced side effects [233]. NR modulators have broad therapeutic applications in cancer, metabolic disorders, and inflammatory conditions.

Ongoing studies in both humans and model organisms continue to reveal how ligand-dependent NR networks integrate hormonal, dietary, and environmental cues to control gene expression in development, metabolism, and disease.

## 10. Pharmacological Approaches to Modulation of Transcription Factor Activity

There are several main strategies by which pharmaceutical agents can regulate TF activity. These approaches are listed below with examples.

### 10.1. Direct and Indirect Small Molecule TF Inhibitors

**Figure 1:**
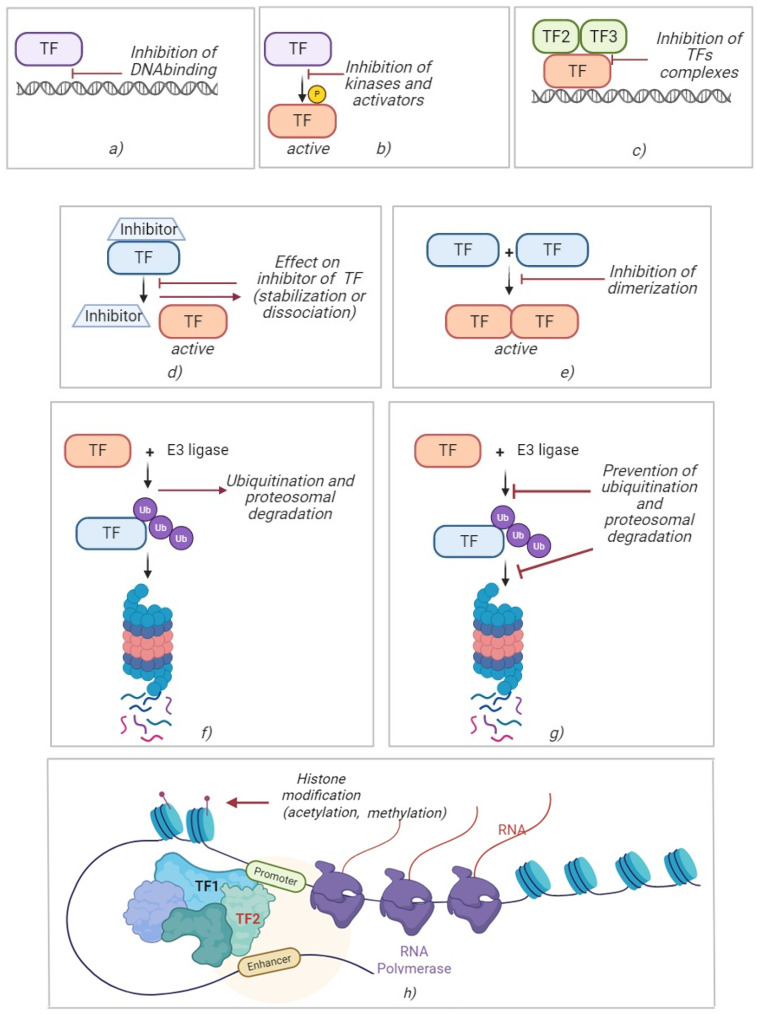
Strategies for modulating transcription factor activity. Inhibition of binding TF with DNA (**a**). Inhibition of TF kinases and activators (**b**). Inhibition of formatting the complex of several TFs (**c**). Stabilization or dissociation of TFs’ inhibitors (**d**). Inhibition of dimerization of TFs (**e**). Ubiquitination and proteosomal degradation of TFs (**f**). Prevention of ubiquitination and proteosomal degradation of TFs (**g**). Histone modification by acetylation or methylation (**h**).

Small molecules can directly and indirectly bind to TFs and block their function. As a rule, they target the DNA-binding domain or key protein–protein interactions. For example, T-5224 is an AP-1 inhibitor that binds to the c-Fos/c-Jun complex and prevents AP-1 from contacting DNA (Figure 1) [234]. Another example is Nutlin-3, which occupies a pocket on MDM2 and prevents MDM2 from binding to p53, thereby releasing p53 from negative control (Figure 1, Table 2) [127,131,132].

There are also molecules that block factor dimerization, for example, acriflavine for HIF-1α (Figure 1) [158,159]. Stereoisomers of delavatin A inhibit SH2 domains 323-1 and 323-2 for STAT3 (Figure 1) [235]. Direct and indirect inhibitors are difficult to develop due to the need for high affinity and specificity to the protein surface, but successful examples prove the feasibility of this approach.

### 10.2. Activators and Restorers of TF Function

Activators and restorers increase the activity of TF factors. Usually these drugs act indirectly, suppressing their inhibitors or stabilizing the TF molecule. For example, dimethyl fumarate, which modifies Keap1, activates the Nrf2 factor [200].

Another example is the MDM2 inhibitors Nutlin and its analogs, which activate the p53 pathway in cells with wild-type TP53 [8]. This also includes the compound APR-246, which chemically “repairs” mutant p53, restoring its ability to bind to DNA [122,236].

Activators are used less frequently than inhibitors, but have a niche value, for example, for neuroprotection, stimulation of regeneration, or destruction of a tumor by restoring the function of a lost suppressor.

### 10.3. PROTAC and Molecular “Glues” (Targeted Degradation)

This constitutes an innovative approach, in which the goal is not to block TF activity, but to completely remove the protein from the cell (Figure 1). PROTACs are bifunctional molecules that bind TF and E3 ubiquitin ligase simultaneously, causing ubiquitination and the subsequent degradation of TF. PROTACs have already been developed for STAT3 (SD-36 compound) and other factors, showing high efficiency and selectivity [189,190,191]. Molecular “glues” are small molecules that change the specificity of endogenous ligases. For example, lenalidomide, pomalidomide, and thalidomide analogs, by binding to Cereblon, induce selective destruction of IKZF1/3 transcription factors in myeloma cells (Figure 1) [237,238,239,240,241].

Molecular “glues” change the specificity of endogenous ligases, causing them to destroy the target protein. An example is the immunomodulators lenalidomide and pomalidomide, which are derivatives of thalidomide. These drugs, used in multiple myeloma, bind to CRBN ligase and redirect it against certain B-lymphocyte transcription factors IKZF1 (Ikaros) and IKZF3 (Aiolos). As a result, lenalidomide causes ubiquitination and selective degradation of IKZF1/3, which are necessary for the survival of myeloma cells, which explains its clinical efficacy (Figure 1) [242,243]. This example illustrates how pharmacological action on TF can be carried out indirectly, through stabilization or degradation of the regulators of these factors. Methods of targeted degradation allow the elimination of “elusive” factors and bypass the problem of functional reserve pathways; the cell simply loses the factor in question. Targeted degradation opens the way to therapeutic intervention on previously inaccessible targets, but requires careful assessment of off-target effects and possible irreversible toxicity.

### 10.4. Epigenetic Modulators

Although epigenetic modulators do not strictly target a single TF, changes in the chromatin landscape and chromosome structure affect the activity of entire TF networks. Histone acetylase (HDAC) or DNA methyltransferase inhibitors can reactivate previously suppressed tumor suppressor genes or reduce oncogene expression by indirectly targeting TFs (Figure 1). For example, the BET bromodomain inhibitor JQ1 blocks the BRD4 protein required for MYC transcription, resulting in a decrease in the level of the c-Myc oncoprotein and the shutdown of a wide range of Myc-dependent genes [244,245]. Thus, JQ1 indirectly “targets” the Myc transcription factor, which was considered non-inhibitable, through an epigenetic mechanism [246].

Another example is the use of HDAC inhibitors in cancer, for example, vorinostat, romidepsin, and belinostat, which change histone acetylation, leading to the activation of differentiation and apoptosis pathways controlled by various TFs [246,247]. Epigenetic drugs often act globally and can cause side effects, but in the context of multifactorial diseases they are useful in combination with more specific agents.

## 11. Challenges, Limitations, and Solutions in Developing TF-Targeted Drugs

Despite progress in the “targeted” modulation of transcription factors, this direction faces a number of problems. First, most TFs are not enzymes, but proteins with a large surface area for interaction with DNA or partners, without deep pockets for a small ligand. Their flexible, often unstructured configuration in the absence of DNA, complicates classical drug design. That is why TFs were long considered “inaccessible” to small molecules [9]. Second, TFs usually control many genes in different cells, a process which is fraught with undesirable effects when inhibited systemically. For example, NF-κB is necessary for normal immune response, so its long-term blockade leads to immunosuppression and increased susceptibility to infections [248]. This limits the therapeutic window for NF-κB inhibitors and requires careful risk/benefit weighing. Similarly, p53 activation in normal tissues can cause damage to hematopoiesis, and Nrf2 upregulation can cause unwanted chemo-resistance in tumors.

Specificity is another important parameter. Many TFs—for example, STAT1-4, NF-κB/RelA-c-Rel, etc.—belong to families of similar proteins, and small molecules may not distinguish between them. However, newer technologies, as shown with PROTAC SD-36, allow achieving high selectivity. SD-36 degraded STAT3 without affecting other STATs [189]. However, there is always a risk that the drug will affect adjacent pathways. For example, JAK inhibitors targeting STAT signaling simultaneously suppress multiple cytokine cascades, going beyond one pathology.

Another complication is cell adaptation and redundancy of signaling pathways. Cancer cells, for example, can bypass the blockade of one factor by activating alternative transcriptional programs. It is known that inhibition of NF-κB in a tumor can be partially compensated by other inflammatory pathways, so combination therapy is required for the effect [73].

In addition, factors can have several functions, encompassing transcriptional and non-transcriptional activity. For example, STAT3 is involved in the work of mitochondria, found in the membranes of the endoplasmic reticulum (MAM) [249,250]. An inhibitor that blocks only the function in the nucleus may leave the other role of the factor intact. The solution may be the complete degradation of the protein, as PROTACs do, or a combination of drugs that eliminate all functions.

Pharmacokinetic aspects, such as delivery to the desired cells, penetration into the nucleus, and stability, are also important, but modern carriers in the form of nanoparticles, liposomes, and conjugates help to overcome these barriers [159]. Off-target toxicity is especially relevant for epigenetic agents that affect global gene expression; such drugs are used with caution and under the control of biomarkers [9].

In general, the development of drugs against TFs requires a balance between efficacy and safety. New methods of computer design, structural biology, and screening allow us to find molecules to target previously “elusive” protein surfaces. Thus, the emergence of three-dimensional structures of complexes for p53–MDM2 and HIF-1α–ARNT directed the synthesis of inhibitors Nutlin, Acriflavine, and others.

However, even knowledge of the structure does not guarantee success. It is critical to take into account the biological context and selectively affect pathological cells. One way to increase specificity is targeted delivery, for example, an antibody-molecular conjugate, where the antibody brings the inhibitor directly to tumor cells expressing a certain TF. Another approach is prodrug forms that are activated only in affected tissues under the influence of enzymes or microenvironmental conditions, such as hypoxia or pH. Challenges and solutions in developing TF-targeted drugs are represented in Table 3.

## 12. Conclusions

Transcription factors are key nodes of gene regulation, and their imbalance underlies many diseases from cancer to autoimmune disorders. Despite the long reputation of “undisclosed” targets, in recent years, convincing examples have emerged of how TF activity can be pharmacologically modulated. Some of these approaches have already been implemented in drugs. For instance, as hormonal antagonists to nuclear receptors, proteasome inhibitors are used for the indirect blockade of NF-κB and thalidomide-type immunomodulators for the degradation of IKZF1/3. Others are under investigation, including direct inhibitors of protein-DNA and protein–protein interactions, molecules for the reactivation of lost functions (p53), as well as a technologically new class of PROTACs that destroy “inaccessible” factors. Combinations of epigenetic agents with targeted TF’s inhibitors are actively studied in order to simultaneously affect several levels of gene regulation.

It is obvious to the scientific and clinical community that the therapeutic targeting of TFs opens up qualitatively new possibilities in the treatment of diseases, but requires a balanced approach. Additional research is needed to improve the specificity of such drugs—for example, the development of molecules that distinguish onco-specific versions of factors in the case of mutant or post-translationally modified forms in cancer cells. An important direction of future work will be monitoring side effects and creating models for predicting the network consequences of intervention in transcriptional networks. Also promising is the development of delivery systems that limit the action of potent TF inhibitors to target tissues only.

In summary, it can be said that the pharmacological correction of transcription factor activity is turning from a concept into a reality of modern medicine. The development of this area encourages interdisciplinary cooperation and the unification of efforts of molecular biologists, chemists, pharmacologists, and clinicians. As a result, this integration of knowledge will help to transform fundamental discoveries about the work of TFs into effective and safe drugs for the treatment of serious diseases.

## Figures and Tables

**Table 1:** Major classes of transcription factors, their functions, and associated diseases.

TF Class/Family	Representatives (Examples)	Key Functions	Associated Diseases	References
NF-κB (Rel)	p65 (RelA), p50, RelB, etc.	Regulation of inflammation and immune response	Autoimmunity, inflammation, cancer	[19,35,36,37,38,39,40,41,42,43,44,45,46,47,48,49,50,51,52,53,54,55,56,57,58,59,60,61,62,63,64,65,66,67,68,69,70,71,72,73,74,75,76,77,78,79,80,81,82,83,84,85,86,87,88,89,90,91,92,93,94,95,96,97,98,99,100,101,102,103,104,105,106,107,108,109,110,111,112,113,114,115,116,117,118,119,120,121,122,123,124]
p53-like	p53, p63, p73	Tumor suppressors, cell cycle and apoptosis control	Cancer (TP53 mutations), degeneration	[125,126,127,128,129,130,131,132,133,134,135,136,137,138,139,140,141,142,143,144,145,146,147,148,149]
STAT (JAK/STAT)	STAT1, STAT3, STAT5, etc.	Cytokine signaling, immune regulation	Inflammation, immune disorders, cancer	[150,151,152,153,154,155,156,157,158,159,160,161,162,163,164,165]
HIF (hypoxia factors)	HIF-1α, HIF-2α (in complex with ARNT)	Adaptation to hypoxia, angiogenesis	Cancer (hypoxia in tumors), ischemia	[166,167,168,169,170,171,172,173,174,175,176,177,178,179,180,181,182,183,184,185,186,187,188,189,190,191,192]
AP-1 (bZIP)	c-Fos, c-Jun, ATF	Stress response, differentiation, proliferation	Inflammation, cancer (stimulation of oncogenes)	[193,194,195,196,197,198,199]
Nrf2 (CNC-bZIP)	Nrf2 (NFE2L2)	Antioxidant response, detoxification	Neurodegeneration, chronic inflammation	[200,201,202,203,204,205,206,207,208,209,210,211,212,213,214,215,216]
Nuclear receptors	ER (estrogen), AR (androgen), PPAR, GR, etc.	Regulation of development and metabolism under the influence of ligands (hormones, lipids)	Cancer (hormone-dependent), metabolic diseases	[217,218,219,220,221,222,223,224,225,226,227,228,229,230,231,232,233]

**Table 2:** Examples of pharmacological agents for modulating transcription factors.

Drug (Mechanism)	Target (TF or Pathway)	Mechanism of Action	Stage of Application
Bortezomib (proteasome inhibitor)	NF-κB (via stabilization of IκB)	Blocks proteasomal degradation of IκBα, preventing NF-κB from entering the nucleus. Indirectly suppresses expression of NF-κB-dependent genes [106].	Approved (multiple myeloma); side effects: immunosuppression [107].
Dexamethasone (glucocorticoid)	NF-κB (indirectly)	NF-κB (indirectly). Induces expression of NF-κB inhibitors (IκBα), inhibits translocation of NF-κB into the nucleus [117].	Widely used as an anti-inflammatory; immunosuppressant, included in the standard treatment for multiple myeloma [118].
Nutlin-3a (small molecule)	p53 (via MDM2)	Binds to MDM2, disrupting the MDM2-p53 interaction, stabilizes p53, and activates its target genes [129,144].	Clinical trials in cancer, including reducing systemic toxicity [129,144,145,146].
APR-246 (PRIMA-1^Met)	p53 (mutant forms)	Metabolized to an electrophilic compound that covalently binds mutant p53, restoring its wild-type conformation [139,140].	Clinical trials (cancer with TP53 mutation) [139].
Acriflavine (small molecule)	HIF-1α	Binds to HIF-1α/2α (PAS-B domain), preventing dimerization with HIF-1β and thereby inhibiting transcription of hypoxia-inducible genes [155].	Preclinical studies; requires toxicity reduction [157].
Roxadustat (PHD prolyl hydroxylase inhibitor)	HIF-1α (stabilization)	Inhibits HIF prolyl hydroxylases, preventing hydroxylation and degradation of HIF-1α; mimics hypoxia by enhancing erythropoietin synthesis [163].	Approved for anemia of chronic kidney disease [163].
Stattic (small molecule)	STAT3	Allosterically binds to the SH2 domain of STAT3, blocks dimerization of STAT3 monomers and their phosphorylation, suppressing STAT3 activity [177,178,179].	Preclinical tool; not used clinically [177,178,179].
Ruxolitinib (JAK inhibitor)	STAT (JAK/STAT pathway)	Competitively inhibits JAK1/2 (ATP node), preventing phosphorylation of STAT1-3. Reduces production of proinflammatory cytokines [185,186,187].	Approved for myelofibrosis, polycythemia, osteoporosis [185,186,187].
SD-36 (PROTAC)	STAT3	Hybrid: contains a ligand to STAT3 and Cereblon; causes ubiquitination and proteasomal degradation of STAT3. Completely eliminates STAT3 from cells [189,190,191].	Preclinical (leukemia and lymphoma models); high efficacy [189,190,191].
T-5224 (small molecule)	AP-1 (c-Fos/c-Jun)	Binds to the AP-1 complex, specifically inhibiting its DNA-binding activity. Reduces expression of cytokines and MMPs [197,198].	Phase II clinical trials (rheumatoid arthritis); not approved [198].
Dimethyl fumarate (electrophile)	Nrf2 (via Keap1)	Covalently modifies Keap1, leading to dissociation of Keap1-Nrf2 and activation of transcription of antioxidant genes [208,209,210,211].	Approved (multiple sclerosis, psoriasis); main effects—immunomodulation [208,209].
Lenalidomide (molecular glue)	IKZF1/3 (IRF family factors, B cells)	Binds to the E3 ligase CRBN, redirecting it against IKZF1/3 factors, causing their selective degradation. Indirectly suppresses Myc, IRF4 gene expression in myeloma [212,213].	Approved for multiple myeloma, lymphoma; also for immunomodulation [212,213].

**Table 3:** Challenges and solutions in developing TF-targeted drugs.

Modality	Challenge	Established Solutions
Lack of ligandable pockets and “undruggable” interfaces	Many TFs are intrinsically disordered or have large, flat protein surfaces without the deep binding pockets that conventional small-molecule drugs require. As a result, although hundreds of disease-associated TFs are known, only a very small number have been successfully drugged with traditional inhibitors. Example: The oncogenic TF c-Myc exemplifies an “undruggable” target: it lacks a stable binding pocket and continuously changes conformation, foiling standard small-molecule design approaches [251].	1. Proteolysis-Targeting Chimeras (PROTACs). These bifunctional molecules recruit an E3 ubiquitin ligase to tag the TF for degradation, bypassing the need for a functional binding pocket. Even TFs that rely on protein–protein or DNA interactions can be eliminated via PROTACs. PROTAC degraders against historically “undruggable” TFs have entered the clinic; for example, TFs in hormone-driven cancers ARV-110 and ARV-471 degrade the androgen and estrogen receptors, and have shown efficacy in trials. PROTACs for TFs involved in cancer and immune diseases are developing [252,253]. 2. Peptide and Protein Inhibitors. Larger biomolecules can be engineered to disrupt TF function by mimicking key interfaces. Omomyc is a 91-amino-acid “mini-protein” designed to mimic Myc’s partner MAX, thereby sequestering Myc in an inactive complex. Omomyc overcomes Myc’s lack of pockets by using a large interaction surface to bind Myc. The purified Omomyc protein was found to inherently penetrate cancer cells, enabling it to be used as a drug. Omomyc (OMO-103) became the first direct Myc inhibitor to reach clinical trials where it showed a favorable safety profile and evidence of target engagement [254]. 3. Epigenetic Cofactor Modulators. An indirect strategy is to target the coactivators or chromatin regulators that TFs depend on. Bromodomain and extraterminal (BET) proteins like BRD4 are “epigenetic readers” that facilitate transcription of oncogenic genes (including MYC). Small-molecule BET inhibitors do not bind Myc itself but block BRD4’s function at super-enhancers, thereby downregulating MYC transcription and other tumor drivers. Several BET inhibitors have entered clinical testing in cancer, demonstrating proof-of-concept that modulating a TF’s epigenetic machinery can achieve a therapeutic effect when the TF itself is intractable. Combination approaches with BET inhibitors are also explored to enhance efficacy, given their limited success as monotherapy [244,255].
Redundancy and Compensatory Pathways	TF often operate in complex, overlapping networks, so cells can compensate for the loss of a single factor. Redundancy in TF families and parallel pathways means that inhibiting one TF may trigger alternative regulators to take over its function. In cancer, for instance, blocking one oncogenic TF can lead tumor cells to upregulate a different TF or signaling route to maintain survival. Similar TF “crosstalk” and backup mechanisms occur in immune diseases, where multiple inflammatory TFs can induce overlapping gene programs and genetic disorders.	1. Combination Therapies: A proven way to address network redundancy is to co-target multiple factors or pathways simultaneously. By inhibiting both the primary TF and its compensatory partner, one can prevent the escape mechanism. For example, preclinical and clinical studies suggest that inhibiting GR in addition to AR can overcome resistance in castration-resistant prostate cancer. GR antagonist (ORIC-101) has been tested in combination with AR pathway inhibitors to block the GR-mediated survival route. More broadly, combination regimens are commonly used in oncology and immunology to shut down parallel pathways. Using multiple targeted drugs or a targeted agent plus chemotherapy/immunotherapy reducing the chance that an alternative transcriptional program can sustain the disease. This strategy has been validated by improved outcomes in cases where single-agent TF targeting failed due to compensation [256]. 2. Broad-Spectrum or Sequential Targeting: In some scenarios, drugs that impact a broader transcriptional program can be employed to avoid narrow targeting of one TF. For instance, inhibiting a critical upstream signaling kinase might simultaneously dampen several TF outputs. While this comes with higher toxicity risk, it can be useful in short bursts or sequentially. Another approach is adaptive therapy: monitoring for pathway changes and then targeting the newly active TF once compensation is detected [257].
Off-Target Effects and Specificity Issues	Achieving high specificity is difficult when targeting transcription factors. Small molecules intended to disrupt a TF’s protein–protein or DNA-binding interactions may unintentionally bind to other proteins or DNA regions, causing off-target effects. Likewise, therapies like DNA decoys or antisense oligonucleotides can have promiscuous actions; for example, a decoy sequence might sequester related TF family members, or exogenous DNA/RNA might activate innate immune sensors. These off-target activities can lead to unintended gene expression changes and toxicities. Ensuring that only the desired TF is affected is a major hurdle. Early TF inhibitors often suffered from poor selectivity, limiting their clinical utility.	1. Targeted Delivery for Specificity minimizes off-target exposure elsewhere. Antibody-drug conjugates (ADCs) carry TF inhibitors or degraders as payloads. By attaching a TF-directed molecule to an antibody against an antigen on tumor or immune cells, the therapy is delivered predominantly to those cells. This approach has been used with a PROTAC whereby an anti-CD33 antibody was conjugated to a PROTAC molecule (BMS-986497) to deliver it specifically into CD33-positive leukemia cells. The result is highly selective TF modulation; the payload (which degrades a transcriptional regulator) is released inside target cells, sparing other tissues. Another delivery-based tactic is using tissue-localized administration. For instance, an oligonucleotide decoy against STAT3 was injected directly into head and neck tumors in a phase 0 trial, which abrogated STAT3’s activity in the tumor with negligible systemic exposure. By localizing treatment to disease sites off-target effects on other organs are greatly reduced [258]. 2. Enhanced Molecular Specificity. Allosteric modulators are designed to bind unique regulatory sites on a TF or its cofactor, distinct from those on other proteins. For example, drugs reactivating mutant p53 (a tumor-suppressor TF) are engineered to bind the mutant conformation of p53, which normal p53 in healthy cells does not adopt, providing a selective effect in p53-mutant tumors. PROTACs themselves can offer a specificity advantage: since a PROTAC must bind both the target TF and a specific E3 ligase, the probability of off-target degradation is lowered. Finally, the use of conditional activation, for example, drugs activated by light or by tumor-specific enzymes, has shown robust preclinical success in restricting a TF inhibitor’s action to particular contexts, thereby reducing off-target impact [191].
Toxicity in Normal Cells and Tissues	On-target effects in normal cells lead to dose-limiting toxicity. Pan-inhibitors of BET bromodomain proteins caused significant hematological toxicity in trials; the most common serious side effect was thrombocytopenia, likely because normal megakaryocytes require BET function for platelet production. This on-target adverse effect has in some cases led to bleeding risks. Other TF-targeted interventions can similarly affect normal proliferative tissues; a broad inhibitor of c-Myc would be expected to impact intestinal crypts and bone marrow, and global NF-κB blockade could suppress needed immune responses. A key difficulty is finding a therapeutic window—a dose that hits the TF in diseased cells but spares enough activity in normal cells to avoid toxicity.	1. Exploiting Differential Dependency. Often, cancer or diseased cells are more reliant on a given TF than normal cells, and this can be leveraged to widen the safety margin. In preclinical models, Myc inhibition via Omomyc had an “extraordinary therapeutic window”: it dramatically halted tumor growth while only slowing the proliferation of normal cells. Tumor cells, which are “addicted” to Myc, underwent apoptosis, whereas normal tissue tolerated partial Myc suppression with reversible effects. This differential dependency allows lower doses or partial inhibition to effectively kill diseased cells. Clinically, careful titration of dose and intermittent dosing schedules are used to maintain efficacy while giving normal tissues time to recover—an approach taken with some BET inhibitors and MYC-targeting agents [259]. 2. Mitigating Toxicity with Combinations. Combining therapies can also mitigate toxicity by enabling lower doses of each agent. A synergistic drug combination can achieve the desired anti-disease effect with each drug at a sub-maximal dose, reducing side effects. For example, preclinical studies showed that pairing BET inhibitors with other treatments allowed dose reductions that minimized overlapping toxicities. Combination regimens are often used, targeting different pathways or adding a less toxic baseline therapy, so that no single drug has to be pushed to a dose that causes intolerable harm. Additionally, selective targeting strategies directly contribute to reduced systemic toxicity by concentrating the drug in diseased tissue. In the case of the PROTAC-ADC example, the toxic effects of the degrader payload on normal cells were diminished by restricting its action to antigen-positive tumor cells. Finally, modern TF-based therapies are engineered to be reversible or tunable; for instance, some gene therapies include safety switches to turn off an introduced TF if needed [260].
Drug Delivery and Bioavailability Challenges	Many TF-targeted therapeutics are large or biologically fragile molecules that struggle to reach their intracellular nuclear targets. Peptides, proteins, siRNAs, and antisense oligonucleotides (ASOs) face delivery barriers: they may be rapidly degraded in the bloodstream, unable to cross cell membranes, or poorly distributed to the relevant tissue. Thus, delivering a TF-directed agent to the right place in the body and into the cell nucleus at therapeutic levels is a major hurdle. Likewise, early protein-based TF inhibitors were thought impractical because of an assumption that they could not penetrate cells.	1. Nanoparticles and Nucleic Acid Chemistry. Advances in drug delivery systems have largely solved the problem of getting nucleic-acid therapies inside cells. Lipid nanoparticles (LNPs) encapsulate siRNA or ASOs and ferry them across cell membranes. Such nanoparticles protect the cargo from degradation and can be targeted to specific organs by modifying their surface. TFs can be inhibited via RNA interference or antisense in vivo, provided an appropriate delivery carrier is used [261,262,263]. 2. Viral Vector Gene Therapy. Instead of delivering a drug to modulate a TF, another approach is to deliver the gene encoding a TF or a corrective version of it directly into cells. Gene therapy vectors can introduce DNA that either expresses a functional TF protein or a regulatory RNA to alter TF activity. A landmark example is Gendicine, a recombinant adenovirus carrying the wild-type TP53 gene. Rather than trying to drug the mutant p53 protein, Gendicine supplies a fresh copy of the p53 transcription factor gene to tumor cells, restoring its tumor-suppressor function [264]. 3. Direct and Targeted Delivery. The therapeutic can be injected or applied locally to maximize concentration at the target for diseases confined to certain sites. Another strategy is to equip therapeutics with cell-penetrating peptides (CPPs) or other delivery tags. In the case of the Myc inhibitor protein Omomyc, it was discovered to have intrinsic CPP-like properties that allowed it to cross cell membranes on its own. This finding enabled systemic administration of a mini-protein that homes to nuclei and blocks Myc. There are also antibody-based delivery systems and exosome-based delivery vehicles under investigation [265].
